# Effect of Temperature on Electrochemically Assisted Deposition and Bioactivity of CaP Coatings on CpTi Grade 4

**DOI:** 10.3390/ma14175081

**Published:** 2021-09-05

**Authors:** Bożena Łosiewicz, Patrycja Osak, Joanna Maszybrocka, Julian Kubisztal, Sylwia Bogunia, Patryk Ratajczak, Krzysztof Aniołek

**Affiliations:** 1 Institute of Materials Engineering, Faculty of Science and Technology, University of Silesia in Katowice, 75 Pułku Piechoty 1A, 41-500 Chorzów, Poland; patrycja.osak@us.edu.pl (P.O.); joanna.maszybrocka@us.edu.pl (J.M.); julian.kubisztal@us.edu.pl (J.K.); patrykrataj@interia.pl (P.R.); krzysztof.aniolek@us.edu.pl (K.A.); 2 Old Machar Medical Practice, 526-528 King Street, Aberdeen AB24 5RS, UK; sylwia.bogunia@nhs.scot

**Keywords:** amorphous calcium phosphate, bioactivity, electrochemically assisted deposition, titanium

## Abstract

Calcium phosphate (CaP) coatings are able to improve the osseointegration process due to their chemical composition similar to that of bone tissues. Among the methods of producing CaP coatings, the electrochemically assisted deposition (ECAD) is particularly important due to high repeatability and the possibility of deposition at room temperature and neutral pH, which allows for the co-deposition of inorganic and organic components. In this work, the ECAD of CaP coatings from an acetate bath with a Ca:P ratio of 1.67, was developed. The effect of the ECAD conditions on CaP coatings deposited on commercially pure titanium grade 4 (CpTi G4) subjected to sandblasting and autoclaving was presented. The physicochemical characteristics of the ECAD-derived coatings was carried out using SEM, EDS, FTIR, 2D roughness profiles, and amplitude sensitive eddy current method. It was showed that amorphous calcium phosphate (ACP) coatings can be obtained at a potential −1.5 to −10 V for 10 to 60 min at 20 to 70 °C. The thickness and surface roughness of the ACP coatings were an increasing function of potential, time, and temperature. The obtained ACP coatings are a precursor in the process of apatite formation in a simulated body fluid. The optimal ACP coating for use in dentistry was deposited at a potential of −3 V for 30 min at 20 °C.

## 1. Introduction

Modification of biomaterials’ surface is one of the most developed areas of material engineering in recent years [1,2,3]. One of the most important challenges is selecting the chemical composition that determines the biocompatibility of the material in the environment of body fluids. The progressive development of civilization requires the design of biomaterials with high durability and supporting the regeneration process. These assumptions are achieved by the deposition of bioactive coatings on the surface of metallic biomaterials. The chemical composition of such biomimetic coatings is similar to that of the surrounding tissues. They create a microenvironment that enables osseointegration and reconstruction of tissues surrounding the implant. Simultaneously, they can be a source of tissue-forming elements and constitute a carrier of medicinal substances.

Titanium and titanium-based alloys are the most commonly used metallic biomaterials due to their excellent mechanical properties, self-passivation, superior corrosion resistance in a biological environment, and unique biocompatibility [4,5,6,7,8]. Adhesion of both soft tissue and bone to the titanium surface was revealed [9,10,11]. Commercially pure titanium (CpTi) is unalloyed titanium with a stable phase structure. CpTi is available in Grade 1, 2, 3, and 4, which differ in the oxygen content [12,13]. The highest strength of CpTi is observed for the Grade 4 (G4), which has the highest oxygen content and is most often used for dental implants [14]. CpTi G4 is the most commonly used for dental implant production due to the mechanical strength of about 550 MPa and Young’s modulus of about 104 GPa [15,16]. To increase the surface development of titanium implants, sandblasting with abrasive particles is often used [17,18]. The optimum surface roughness of 1–3 µm is obtained when sandblasting with white Al_2_O_3_ particles with a size of 25–75 μm [18].

One way to improve the osseointegration process is the use of biomimetic coatings with a composition similar to that of bone tissues. The best available material is CaP bioceramics, which improves the coating’s bonding on the implant surface with the bone tissue [19,20,21,22]. CaP bioceramics is widely used in medicine, including orthopedics, plastic surgery, and odontology. It is similar to the biogenic CaP found in the inner ear of embryonic sharks, mammalian milk, and dental enamel. Bioactive CaP coatings on the surface of implant can show intrinsic osteoinduction understood as their ability to trigger bone formation in heterotropic sites. Surface composition and both volume and architecture of the CaP coatings play a key role in the process of intrinsic osteoinduction. Bohner and Miron [23] proposed a mechanism of material-induced heterotropic ossification. They reported that the origin of internal osteoinduction is the local consumption/depletion of calcium and phosphate ions through the formation of apatite (Ap). Then, an extracellular matrix is formed on the implant surface, inducing bone tissue regeneration [19,20]. The main component of the mineral substance of the tooth is CaP. Therefore, the use of CaP coatings on the surface of implants will positively affect the osteo-regeneration processes. In addition, CaPs are used in regenerative medicine, in the processes of controlled tissue regeneration, and as scaffolds in drug controlled release systems [19]. There are different types of CaPs depending on the molar ratio of Ca to P.

The difference between CaPs comes from where the phosphate occurs in the body. In dentin, the Ca:P molar ratio is below 1.67, while, for bone tissue, it is above 1.67. In medicine, many crystallographic forms of CaP are used. However, the most popular are the salts of the tribasic orthophosphoric acid in the form of hydroxyapatite (HA), α-tricalcium phosphate (α-TCP), and β-TCP [19]. CaPs, mainly amorphous calcium phosphate (ACP), HA, and α-TCP, are used in dentistry to reconstruct tooth hard tissues as temporary and permanent fillings. ACP and octacalcium phosphate (OCP) during bone tissue remodeling transform into the biological phase. Each CaP is characterized by a different solubility depending on the stoichiometry [19,20].

ACP is a precursor of HA and is involved in the transition phase of mineralization, particularly in the dentin matrix protein (DMP1) [21]. It is a unique form of CaP minerals found in organisms. In the atomic structure of ACP, there is long-range disorganization of crystalline CaPs. It is widely used in dentistry due to its excellent bioactivity and adjustable biodegradation rate. ACP has also been shown to increase mesoblast alkaline phosphatase activity, improve cell proliferation, and promote cell adhesion [24]. Currently, ACP is used in dentistry as a filler in ionomer cements, supports the remineralization process, and prevents the formation of carious lesions [24,25]. ACP neutralizes the acids produced by plaque bacteria [26,27,28]. ACP phases with a Ca:P molar ratio greater than 1.5 are only obtained in the presence of foreign ions, usually carbonate or oxide. Oxide ions in the ACP phase are obtained during the plasma application of HA to the implant surface [29]. When heated, ACP gradually loses water and then transforms into individual crystallographic forms of CaPs. In an acidic environment in the body, ACP releases calcium and phosphorus ions, and then, during crystallization, it binds to collagen fibers [30]. ACP clinical trials show that this material has good properties to convert into bone apatite in vivo, making it an excellent new class material for bone defect replacement and repair [29]. ACP can be combined with antibacterial preparations such as silver. Studies have shown that, in the form of nanoparticles, ACP has intrinsic antimicrobial activity [31].

CaP bioceramics, as bulk implant material under high physiological stress, are limited due to their poor mechanical properties such as brittleness, low fracture toughness, and hardness. Therefore, it is proposed to solve this problem by depositing CaP coatings on biocompatible substrates that can achieve both the necessary mechanical strength and the materials’ bioactive properties [19,32,33,34]. So far, thin CaP coatings have been obtained using various methods, including pulsed laser deposition, ion beam techniques, sputter deposition, electrochemically assisted deposition (ECAD), electrospraying, electrophoretic deposition, biomimetic deposition in simulated body fluid (SBF), and sol–gel method [19]. Recently, arc plasma has been applied to produce bioactive and non-toxic manganese-substituted α–TCP coatings on Ti [35]. These Mn–TCP coatings were composed of α-Ca_3_(PO_4_)_2_ (67%) and HA (33%). They contained 2.3 wt% of Mn and exhibited activity for the proliferation of the human tooth postnatal dental steam cells. The deposition conditions are currently sought for the production of CaP coatings at low temperatures [36,37]. The ECAD method’s production of thin CaP coatings is the most widely used method of surface modification of medical implants. The biomimetic ECAD method is highly reproducible and economical. It provides the ability to deposit thin CaP coatings in aqueous solutions with a neutral pH on electrically conductive substrates and complex shapes. The deposition process can be carried out at room temperature with easy control of the coatings’ thickness and chemical composition down to the submicrometer level. The ECAD method requires a much shorter time due to more defined and higher relative supersaturation at the interface in comparison with the SBF’s biomimetic incubation [19]. The potentially low adhesive strength of the obtained CaP coatings can be overcome by combining the growth of oxides as an intermediate layer on the substrate surface or heat treatment process. For the substrate’s cathodic polarization, constant cell voltage, constant potential, or constant current can be used. Biomimetic CaP coatings such as HA, OCP, ACP, and dicalcium phosphate dihydrate (DCPD), also called brushite, were obtained using the EACD method [19,32,33,34].

The structure and surface morphology of the CaP coatings can be tailored through the selection of conditions of the ECAD process, including electrolyte composition, pH of the electrolyte, temperature, electrochemical conditions, and time of deposition [19,32,33,34,35,36,37,38,39,40,41]. The ECAD method produces the CaP coatings in the amorphous state, and heat treatments at high temperatures are used to achieve high crystallization [38]. The ECAD process’s operating parameters primarily affect stoichiometry, the grade of crystallization, particle size, and microstructure of the obtained CaP coatings, and thus allow to shape their properties as thermal stability, mechanical performance, biocompatibility, and corrosion resistance [39]. The CaP coatings have been deposited so far from a nitrate bath without or with HCl’s addition, which caused the dissolution of individual components of the bath [19,32,33,34,35,36,38,40]. However, the obtained coatings were characterized by a heterogeneous structure. The chloride bath has also been used to obtain the CaP coatings [37,41]. Dilute sodium chloride solution was added to improve the conductivity of the bath [41]. The CaP coatings deposited in the chloride bath at near-physiological conditions with a pH of 6.4 and temperature of 36 °C were characterized by chloride presence [37]. Co-deposition of chloride with CaP coating on the surface of titanium is an unfavorable phenomenon. Chlorine is responsible for normalizing the acid–base and water–electrolyte balance. If the balance between acidity and alkalinity is disturbed by the excess chloride, life processes in the body go wrong.

We have recently reported preliminary results about the possibilities of producing ACP coatings on CpTi G4 using ECAD in a neutral acetate bath with a Ca:P ratio of 1.67 [42]. In this study, we continue our interest in the ECAD-derived ACP coatings towards their dental applications. The influence of the ECAD process parameters on the physicochemical properties of the obtained ACP coatings and their deposition mechanism was studied. In vitro bioactivity as a key quality criterion for the newly developed ACP coatings was determined as a deposition temperature function.

## 2. Materials and Methods

### 2.1. Material Preparation

CpTi G4 was used as a substrate for the ECAD of CaP coatings. Composition limits followed the specifications defined in standards ASTM F67-13 [12] and ISO 5832-2 [13]. Five-mm-high disc-shaped specimens were cut from a 10-mm diameter bar (Bibus Metals, Dąbrowa, Poland). CpTi G4 specimens were subjected to mechanical polishing with 600 to 5000 # grit silicon carbide paper and colloidal silica suspension for finishing polishing with a grain size of 0.04 mm (OP-S suspension, Struers, Cleveland, OH, USA). Polished specimens with a mirror surface were sonicated twice for 20 min, first in acetone and then in ultra-pure water with a resistivity of 18.2 MΩ cm at 25 °C produced by the Milli-Q Advantage A10 Water Purification System (Millipore SAS, Molsheim, France). The CpTi G4 specimens prepared in this way were sandblasted using white Al_2_O_3_ of FEPA Grit F220 [43]. A pressure of 0.6 MPa was used. The sandblasting time was 15 s. The distance of the sand nozzle from the titanium surface was about 1.5 cm. CpTi G4 specimens were washed in an ultrasonic cleaner for 20 min, successively in acetone, and then in ultrapure water to remove corundum residues from sandblasted surfaces. In the next stage, the sandblasted specimens were sterilized in distilled steam at 134 °C under a pressure of 2.2 bar for 90 min using a Zealway Model GR60 DA autoclave (Xiamen, China).

### 2.2. ECAD Conditions

The CaP coatings were deposited on the surface of CpTi G4 subjected to sandblasting and autoclaving using the ECAD in the acetate bath with the following chemical composition [g dm^−3^]: C_4_H_6_CaO_4_—2.94, (NH_4_)_2_HPO_4_—1.32, C_6_H_8_O_7_—2.00, and NH_4_Cl—2.00. This content of the bath components resulted in a Ca:P ratio of 1.67. The addition of C_6_H_8_O_7_ improved the bath components’ solubility, while NH_4_Cl increased the solution’s conductivity. The deposition bath’s pH was adjusted to 7.0(1) with a 30% NH_3_ solution using a CP 101 Elmetron pH-meter. All solutions were prepared from reagents of recognized analytical grade (Avantor Performance Materials Poland S.A., Gliwice, Poland) and ultra-pure water as a solvent.

The CaP coatings were deposited using a three-electrode system in which titanium was the working electrode (WE), platinum was the counter electrode (CE), and the reference electrode was a saturated calomel electrode (SCE, Hydromet, Gliwice, Poland) type R-20 introduced into the system using a Luggin capillary. The WEs with a surface area of 0.785 cm^2^ were obtained by affixing an insulated copper wire to the back of the CpTi G4 specimens with an epoxy resin, which provided electrical contact. The back and sides of the WEs were protected with a chemically resistant, two-component epoxy resin. The surface area of the CE in the form of the mesh was 10 cm^2^. The conditions of the potentiostatic deposition of CaP coatings on the CpTi G4 substrate from the acetate bath were selected based on cathodic polarization curves recorded in the range of potentials from a stationary potential (*E*_stat_) to *E* = −10 V at room temperature. The sweep rate of polarization was *v* = 1 mV s^−1^. The WEs were subjected to chemical activation for 5 min in a 4% aqueous NaOH solution before deposition. Then, the open-circuit potential (*E*_OC_) of the WE was stabilized for 30 min in the acetate bath. Next, the CaP coatings were potentiostatically deposited at a potential value ranging from −1.5 to −10 V, relative to the *E*_OC_ for 10 to 60 min. The temperature of the deposition bath was varied from 20 to 70 °C. Electrochemical measurements were carried out using the potentiostat Autolab/PGSTAT30 (Metrohm Autolab B.V., Utrecht, The Netherlands). After the ECAD process was complete, the WEs were rinsed thoroughly with ultrapure water and dried in air at room temperature for 1 h until the CaP coatings turned white. The weight gain was determined from the difference in weight before and after the deposition of the coatings. The numerical values of the standard uncertainty referred to the corresponding last digit of the quoted result were given in parentheses [44].

### 2.3. Physicochemical Characteristics of CaP Coatings

The thickness of the obtained CaP coatings was determined using a Dualscope FMP20 gauge (Helmut Fischer GmbH, Sindelfingen, Germany), equipped with a probe FTA 3.3 (Helmut Fischer GmbH, Sindelfingen, Germany) and amplitude-sensitive eddy current method based on the electromagnetic induction phenomenon. The probe used for measuring had a ferrite core on which the coil was wound. An alternating current flowing through the coil generated an alternating magnetic field around it. When the probe was brought close to a conductive material, the eddy current was then induced in it. The eddy current flowing through the material generated its magnetic field, which sup-pressed the one generated by the coil. The extent of the attenuation was dependent on the distance between the probe and conductive material (substrate), i.e., on the coating thickness. Since the amplitude-sensitive eddy current method is a comparative technique, the device before measurements had to be calibrated to the specific coating/substrate system. Calibration was conducted using the uncoated titanium substrate and two standard foils with a thickness of 24.3(5) and 48.2(1) μm (Helmut Fischer GmbH, Sindelfingen, Germany).

The surface morphology and local chemical composition were examined using the JEOL JSM-6480 scanning electron microscope (SEM, Peabody, MA, USA), equipped with an energy dispersion spectroscopy (EDS) attachment.

The surface roughness of the tested materials was studied using the Mitutoyo Surftest SJ-500/P profilometer. The surface profile changes were measured with a measuring step of 0.1 μm and a speed of 200 μm s^−1^, over a length of approx. 10 mm. Recorded parameters according to ISO 4287 [45] were processed and developed using the FORMTRACEPAK computer program.

### 2.4. In Vitro Bioactivity of CaP Coatings

In vitro bioactivity studies were carried out on sandblasted and sterilized CpTi G4 samples before and after the deposition of the CaP coatings. CaP coatings were obtained by ECAD at −3 V for 30 min using a bath temperature of 20 to 70 °C. Five samples were tested in each series. The ability of the samples to form Ap in the SBF was tested according to the procedure proposed by Kokubo and Takadama [46]. Each sample was soaked in 30 mL of acellular SBF at 36.6(1) °C for 1, 3, and 7 days. The concentrations of SBF ions were similar to those in human plasma. The composition of the SBF solution used for the bioactivity test is shown in Table 1.

The SBF solution was prepared by dissolving recognized analytical grade reagents (Avantor Performance Materials Poland S.A., Gliwice, Poland) in ultra-pure water in the following order: NaCl, NaHCO_3_, KCl, K_2_HPO_4_·3H_2_O, MgCl_2_·6H_2_O, CaCl_2_, and Na_2_SO_4_. Tris-hydroxymethylaminomethane (CH_2_OH)_3_CNH_2_ and 1 M of HCl were used to adjust the SBF pH to 7.4(1). The bioactivity test was performed in plastic containers with a smooth surface and without scratching to avoid Ap nucleation induced on the surface or edge of scratches. After soaking, the samples were carefully removed from the SBF, rinsed gently in ultra-pure water, and dried in the open air for 24 h. SEM/EDS and attenuated total reflection—Fourier transform infrared spectroscopy (ATR−FTIR) were used to confirm the formation of Ap on the surface of the samples. The ATR−FTIR absorption spectra were collected in the range of wavenumber 4000–450 cm^−1^ using the Shimadzu IR Prestige-21 FTIR spectrophotometer (Kyoto, Japan) equipped with ATR attachment (diamond n = 2.4 and a beam penetration depth of 1000 cm^−1^).

## 3. Results and Discussion

### 3.1. Potentiodynamic Characteristics of CaP Coating Formation

**Figure 1:**
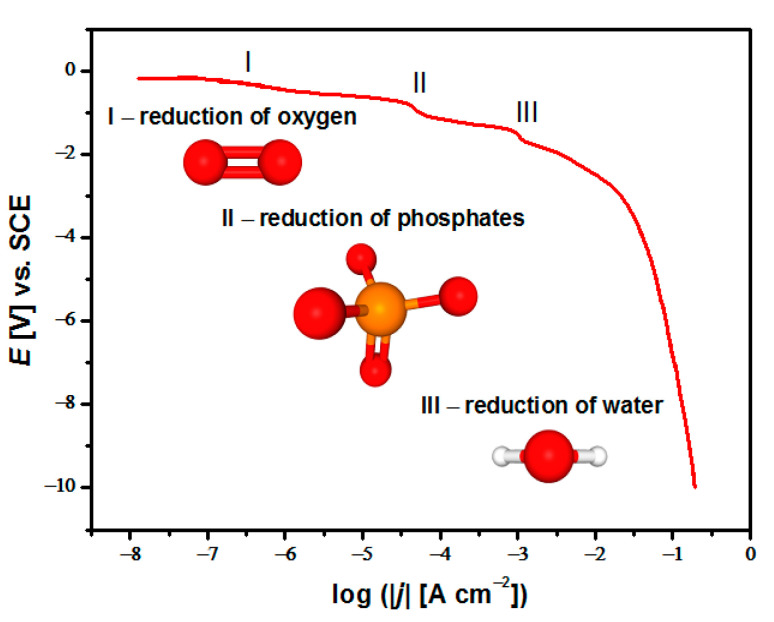
The *E* = f(log|*j*|) polarization curve of the ECAD of the CaP coating on the CpTi G4 substrate in the acetate bath in the potential range from *E*_stat_ to *E* = −10 V vs. SCE at room temperature using *v* = 1 mV s^−^^1^.

The potentiodynamic characterization of the CaP coating formation onto the sandblasted and autoclaved CpTi G4 substrate included the registration of deposition polarization curves in the range of potentials from *E*_stat_ to *E* = −10 V at *v* = 1 mV s^−1^ (Figure 1). Recording of the polarization curve was carried out from the acetate bath at room temperature.

The polarization curve of the deposition of the CaP coating as a dependence of *E* = f(log|*j*|) reveals the presence of three stages, where the first stage (I) at the potential of about −0.6 V is assigned to the reduction in oxygen (1) [41,47,48]:

(1) $$O_{2}+2H_{2}O+4e^{-}\to 4{\mathrm{OH}}^{-}.$$

The second stage (II) in the potential range from −0.6 to −1.6 V corresponds to phosphates’ reduction (2) and (3):

(2) $$2H_{2}{\mathrm{PO}}_{4}^{-}+2e^{-}\to 2{\mathrm{HPO}}_{4}^{2-}+H_{2}↑,$$

(3) $${\mathrm{HPO}}_{4}^{2-}+2e^{-}\to 2{\mathrm{PO}}_{4}^{3-}+H_{2}↑.$$

Stage (III) at cathodic potentials more than −1.6 V is associated with the reduction in water (4) [37,49]:

(4) $$2H_{2}O+2e^{-}\to H_{2}↑+2{\mathrm{OH}}^{-}.$$

The Ca^2+^ ions migrated from the acetate bath volume to the negatively charged cathode surface. They reacted with the ${\mathrm{PO}}_{4}^{3-}$ ions formed in stage II, resulting in CaP synthesis on the CpTi G4 surface. Based on the obtained polarization curves, potentials corresponding to stage III in the range from −1.5 to −10 V were selected for the ECAD of CaP coatings.

**Figure 2:**
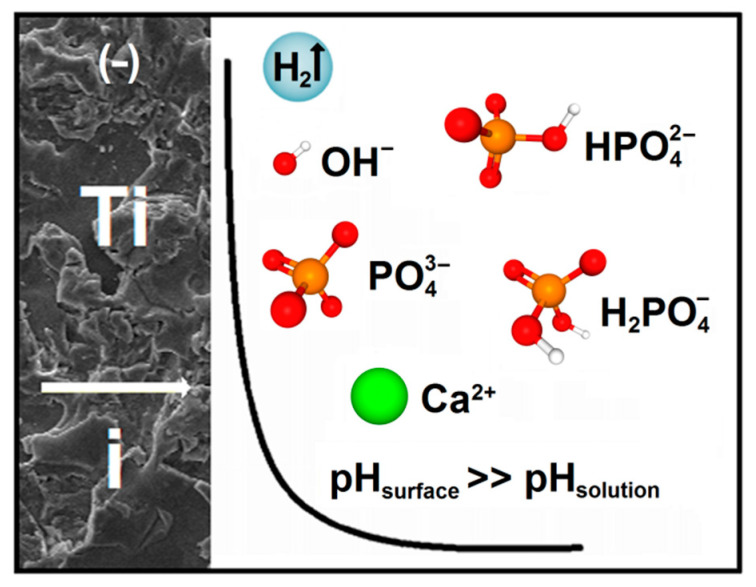
The ECAD mechanism of CaP coatings on the CpTi G4 substrate in the acetate bath.

The ECAD mechanism of CaP coatings on the CpTi G4 substrate in the acetate bath is schematically shown in Figure 2.

In this mechanism, cathodic polarization of the electrically conductive substrate leads to OH^−^ ions formation. As a result of Reactions 1 and 4, there is a local increase in pH at the cathode surface. The pH near the titanium electrode takes much greater values than the pH in the bath volume. Since the solubility of CaP depends on the bath pH, the increase in pH results in an increase in the relative supersaturations of the bath in relation to CaP, leading to the formation of CaP particles. In the ECAD process, electrochemical reactions play a crucial role. Still, it should be noticed that, during the CaP formation, there is no charge carrier transfer as it is in the electrochemical reaction taking place during the electrodeposition of metallic coatings. The ECAD method also differs from the electrophoretic deposition of CaP coatings, in which micro- or nanoparticles of CaP are pre-added to the bath and then deposited on the metallic substrate under an applied electric field.

It was reported in the literature that during the ECAD process, different crystallographic forms of CaP could be obtained (5)–(8) [19,32,33,34,35,36,37,38,39,40,41]:

(5) $${\mathrm{Ca}}^{2+}+H_{x}{\mathrm{PO}}_{4}^{(3-x)-}+(x-1){\mathrm{OH}}^{-}+(3-x)H_{2}O\to {\mathrm{CaHPO}}_{4}\cdot 2H_{2}O\cdot (\mathrm{DCPD}),$$

(6) $${\mathrm{Ca}}^{2+}+6H_{x}{\mathrm{PO}}_{4}^{(3-x)-}+(6x-2){\mathrm{OH}}^{-}\to {\mathrm{Ca}}_{8}H_{2}{{(\mathrm{PO}}_{4})}_{6}+(6x-2)H_{2}O\cdot (\mathrm{OCP}),$$

(7) $${10\mathrm{Ca}}^{2+}+6H_{x}{\mathrm{PO}}_{4}^{(3-x)-}+(6x+2){\mathrm{OH}}^{-}\to {\mathrm{Ca}}_{10}{{(\mathrm{PO}}_{4})}_{6}{\left(\mathrm{OH}\right)}_{2}+6xH_{2}O\cdot (\mathrm{HA}),$$

(8) $$3{\mathrm{Ca}}^{2+}+2H_{x}{\mathrm{PO}}_{4}^{(3-x)-}+2x{\mathrm{OH}}^{-}\to {\mathrm{Ca}}_{3}{\left({\mathrm{PO}}_{4}\right)}_{2}+2xH_{2}O\cdot (\mathrm{ACP}).$$

The indexes and factors *x* in Reactions (5)–(8) are dependent on the bath concentration and indicate that OH^−^ ions’ consumption is a measure of the pH effect on the individual CaP formation. The deposited CaP phases’ composition can be affected by many parameters of which bath composition, temperature, and polarization method play the most important role [37].

**Figure 3:**
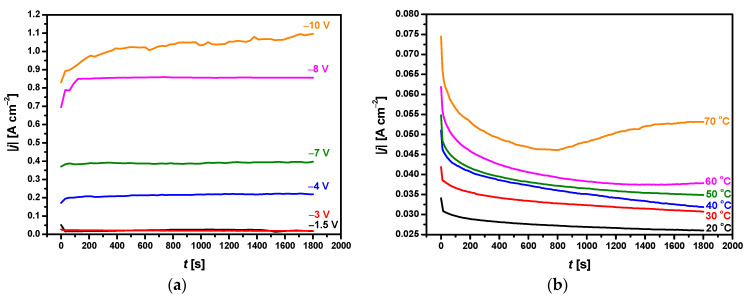
Chronoamperometric curves for ECAD of the CaP coatings on the CpTi G4 substrate in the acetate bath as a function of: (**a**) Deposition potential at room temperature; (**b**) Bath temperature at the deposition potential of −3 V.

Figure 3 depicts chronoamperometric curves recorded during ECAD of the CaP coatings on the CpTi G4 substrate in the acetate bath under selected deposition potentials.

**Figure 4:**
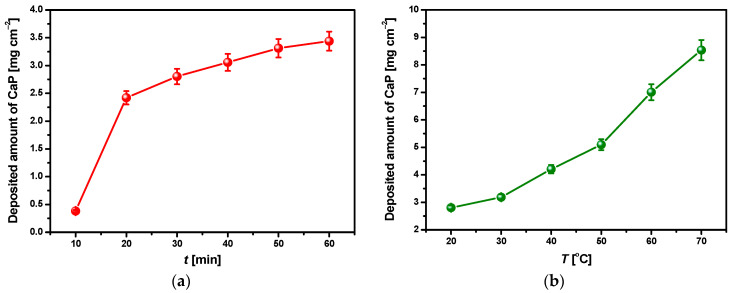
The deposited amount of CaP on the CpTi G4 substrate after ECAD in the acetate bath at the deposition potential of −3 V as a function of: (**a**) Deposition time at room temperature; (**b**) Bath temperature for the deposition time of 1800 s.

The sharp decrease in the value of the current density modulus in the initial stage of the ECAD during the first 30 s is related to the charging of the electric double layer or the rapid formation of the CaP barrier layer on the CpTi G4 surface. The second stage shows the increase in the |*j*|, which can be attributed to the CaP phase’s nucleation at the cathode surface. In the last step of the ECAD, a constant value of |*j*| is reached, which corresponds to the CaP coating’s growth at the CpTi G4 surface at the same deposition rate. Each step duration depends on the applied potential and temperature of ECAD. As shown in Figure 3a, the current density modulus value strongly depends on the deposition potential. The value of |*j*| after 1800 s of ECAD increases from 0.018(3) A cm^−2^ at −1.5 V to 1.096(9) A cm^−2^ at −10 V. The |*j*| = f(*t*) curves recorded as a function of the bath temperature after 1800 s of ECAD at the deposition potential of −3 V show that the value of |*j*| increases from 0.026(3) A cm^−2^ at 20 °C to 0.053(5) A cm^−2^ at 70 °C (Figure 3b). Such a current behavior is probably caused by the increase in bath conductivity with increasing temperature. Similar chronoamperometric characteristics for the ECAD process of CaP coatings on Ti and its alloys from nitrate and chloride baths under a potentiostatic control were reported in the literature [40,41]. The deposited amount of CaP on the CpTi G4 substrate after ECAD at the deposition potential of −3 V at different deposition times and bath temperatures was determined gravimetrically from the mass increase during ECAD (Figure 4).

Figure 4a shows that the amount of CaP deposited on the cathode surface in the acetate bath at the deposition potential −3 V at room temperature increases from 0.382(19) to 3.439(172) mg cm^−2^ after 10 and 60 min of ECAD, respectively. The obtained relationship between the deposited amount of CaP and the deposition time is not linear as it was reported for potentiostatic electrodeposition of CaP on Ti6Al4V alloy in the acidic bath obtained by mixing 1 dm^3^ 0.042 M Ca(NO_3_)_2_ and 1 dm^3^ 0.025 M NH_4_H_2_PO_4_ solutions [40].

The authors stated that the amount of CaP electrodeposited at the potentials of −1.6 and −2.0 V was a function of the deposition time’s square root. The obtained results indicate that the ECAD is limited by diffusion processes that directly affect CaP deposition rate. The increase in the current density causes a faster increase in CaP and, consequently, higher masses of the coatings. The masses of CaP coatings deposited from acidic electrolytes for 1 h are usually 2 to 3 mg cm^−2^ and are comparable with the masses of coatings deposited from neutral electrolytes under hydrothermal conditions [37]. It was reported that the CaP coatings deposited from neutral electrolytes at room temperature feature much lower masses of about 0.1 mg cm^−2^, which are increased by increasing the ECAD time. It should be noted that, in the case of the newly developed acetate bath, the CaP coating characterized by a homogeneous and continuous structure and a mass equal to 2.803(140) mg cm^−2^ is obtained after just 30 min of the ECAD at the potential of −3 V (Figure 4a). The thickness of such a CaP coating is 11.3(7) μm. As the temperature of the ECAD process increases, the mass of this CaP coating increases non-linearly to 8.536(369) mg cm^−2^ at 70 °C, which corresponds to the thickness of 34.4(8) μm (Figure 4b). The obtained results confirm that the increase in the ECAD process’s temperature increases the rate of CaP deposition due to increasing the bath’s conductivity, which results in a significant rise in the masses of the deposited coatings. Ban and Maruno [50] performed a hydrothermal-electrochemical deposition of hydroxyapatite on titanium at higher temperatures ranging from 80 to 150 °C. Furthermore, they showed that the increase in the deposition temperature has a strong effect on the deposition rate. The mass of the coating produced under galvanostatic control at 150 °C was four times greater than that at 80 °C of about 12 mg for a 20 × 20 × 0.5 mm titanium cathode.

### 3.2. Microstructure Studies of CaP Coatings

**Figure 5:**
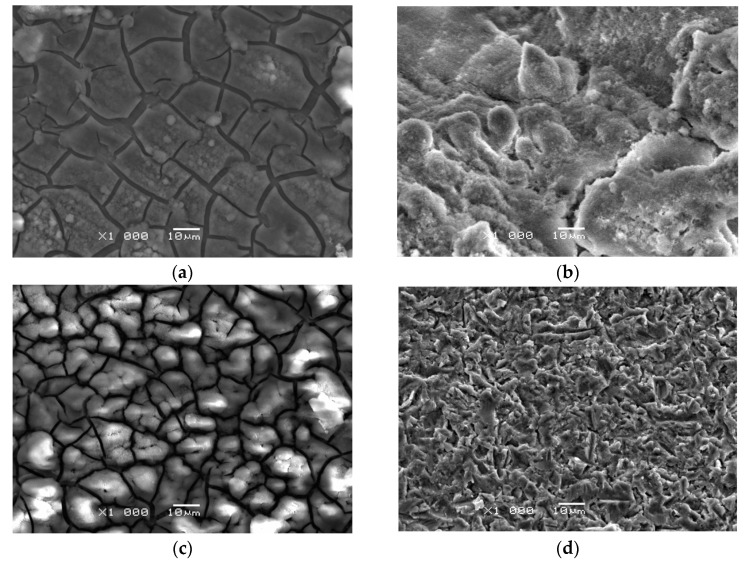
SEM image of the surface morphology: (**a**) CaP coating deposited at −1.5 V for 30 min at 20 °C with a thickness of 0.7(1) μm; (**b**) CaP coating deposited at −3 V for 30 min at 20 °C with a thickness of 11.3(7) μm; (**c**) CaP coating deposited at −10 V for 30 min at 20 °C with a thickness of 20.1(9) μm; (**d**) CpTi G4 substrate.

Rising deposition potential, time, and bath temperature increase the deposited amount of CaP and the produced coatings’ surface morphology. Figure 5 shows SEM images of the CpTi G4 substrate’s surface morphology before and after ECAD of the CaP coatings carried out in the acetate bath at 20 °C for 30 min at different deposition potentials. One can observe that the CaP coating’s microstructure obtained at −1.5 V is characterized by numerous micro-cracks extending to the titanium substrate (Figure 5a). The obtained coating is smooth and thin and shows very poor adhesion to the porous substrate (Figure 5d), making it a non-functional coating. Regardless of the deposition time at such a low potential, no continuous CaP coatings are obtained from the acetate bath. The ECAD carried out at the optimum value of −3 V ensures a thicker and more continuous CaP coating, which completely covers the substrate and adheres well to the titanium’s surface (Figure 5b). The surface morphology of this coating is more developed with minor micro-cracks present. Note that no absolutely dense CaP coatings can be obtained [37]. Increasing the deposition potential to −10 V produces a thick but cracked CaP coating (Figure 5c). The obtained coating shows poor adhesion to the substrate and crumbles easily, which is caused by the intensive co-evolution of hydrogen gas during the ECAD process, according to Reaction 4. Additionally, deposition of the CaP coating at such a high potential for long deposition times can lead to the titanium substrate’s hydrogen embrittlement, capable of hydrogen absorption.

**Figure 6:**
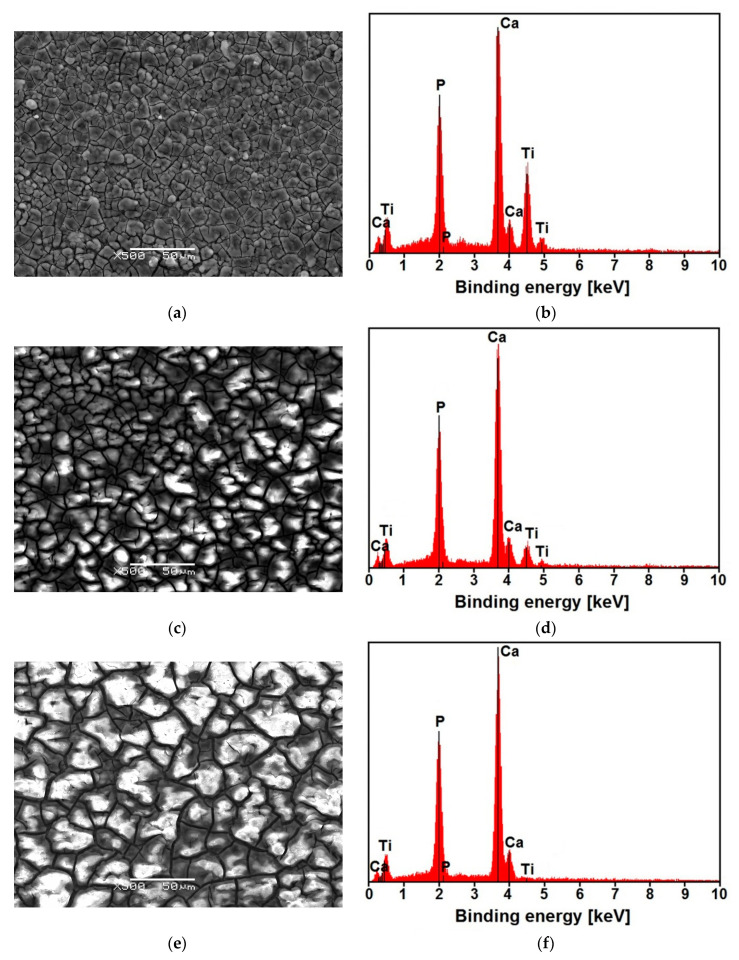
SEM image of the surface morphology with the corresponding EDS spectrum for the CaP coating deposited at −10 V at 20 °C for: (**a**,**b**) 20 min; (**c**,**d**) 30 min; (**e**,**f**) 40 min.

Effect of deposition time on the surface morphology of the CaP coating is presented in Figure 6 for the exemplary coating deposited at −10 V for 20, 30, and 40 min at 20 °C. With such high potential and a short deposition time of 20 min, a fine crystalline coating with an irregular grain shape and various diameter sizes is obtained (Figure 6a). The element concentration determined from the EDS peaks in at.% was: 41.88(16) for Ca, 28.17(38) for P, and 29.95(68) for Ti (Figure 6b). With the increase in the deposition time, the grain size increases, and a coarse-grained structure with a clear mesh of micro-cracks all over the surface is visible (Figure 6c,e). As the ECAD time lengthens, the micro-cracks become more intense, and the coating becomes more brittle due to HER’s adverse process. It can be expected that, as the grain size increases, the properties of the deposited CaP coatings, such as corrosion resistance and mechanical properties, will deteriorate. The element concentration in at.% determined from the EDS spectra was: 45.76(79) for Ca, 30.71(22) for P, and 23.53(13) for Ti (Figure 6d), and 50.19(81) for Ca, 33.69(23) for P, and 16.12(11) for Ti (Figure 6f). Regardless of the deposition time, EDS analysis of the results shown in Figure 6b,d,f showed the Ca:P ratio of 1.5, indicating the presence of ACP [29]. The obtained results are consistent with the CaP electrodeposition mechanism according to Reaction 8.

Based on the broad and diffuse X-ray diffraction pattern with a maximum at 2*θ* of 30, the presence of the Ca_9_O_7_P_2_·H_2_O phase was identified in the CaP coatings on the CpTi G4 surface deposited from an acetate bath [51]. The obtained results are consistent with the EDS data in Figure 6b,d,f. The long-range, periodic atomic-scale order of crystalline CaPs does not exist for ACP. In aqueous media under physiological pH and temperatures, ACP easily hydrolyzes to form octacalcium phosphate as an intermediate, and then surface apatite. ACP also reveals better in vivo osteoconductivity and biodegradability in comparison with tricalcium phosphate and hydroxyapatite [52].

The obtained results confirm that, by using the developed acetate bath with a neutral pH, it is possible: (i) to obtain thin coatings of CaP with a homogeneous structure; (ii) to avoid chloride co-deposition; (iii) to avoid the need to sinter CaP coatings with the substrate; and (iv) to ensure the possibility of co-depositing CaP with other inorganic and/or organic components.

### 3.3. Effect of Deposition Temperature on Surface Morphology and Roughness of CaP Coatings

**Figure 7:**
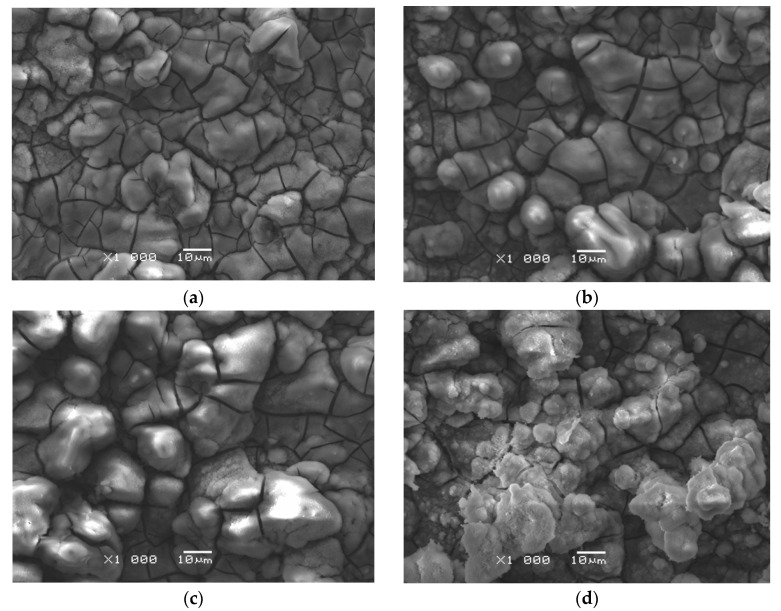
SEM image of the surface morphology for the CaP coating deposited at −3 V for 30 min at: (**a**) 20 °C; (**b**) 30 °C; (**c**) 40 °C; (**d**) 70 °C.

The microscopic observations showed the effect of ECAD’s temperature conditions on the surface morphology of the CaP coatings. Figure 7 presents SEM images of the surface morphology for the CaP coating deposited from the acetate bath at −3 V for 30 min at the bath temperature of 20–70 °C.

The obtained coatings show good adhesion to the porous titanium substrate. The rising temperature of the deposition bath not only increases the deposited amount of CaP (Figure 4b) but also favors the formation of larger crystallites. The obtained results are consistent with the literature data reported by Ban and Maruno [50]. As the bath temperature rises, a more island-like surface structure becomes apparent. As the deposition temperature increases, the tendency of the CaP coatings to form micro-cracks growths can also be observed. This fact may be related to the release of hydrogen bubbles during ECAD. On the coating surface obtained at the temperature of 70 °C (Figure 7d), conglomerates composed of CaP grains are present, which makes that the surface development is slightly smaller than at lower temperatures. The distribution of the fine CaP grains in the conglomerate is quite homogeneous. It means that an increase in the coatings’ deposition temperature favors the coagulation phenomenon consisting of the joining of CaP particles into larger agglomerates.

**Figure 8:**
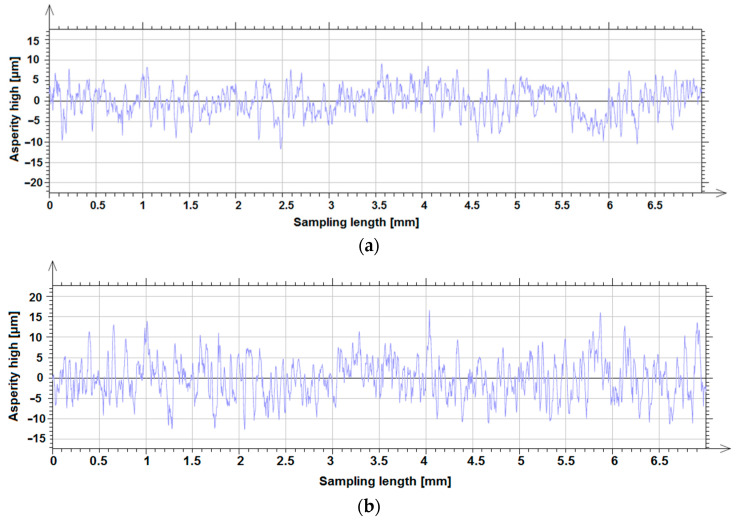
Roughness profile recorded for the CaP coating deposited on the CpTi G4 substrate from the acetate bath at −3 V for 30 min at: (**a**) 20 °C; (**b**) 70 °C.

The effect of deposition temperature on the deposited CaP coatings’ surface roughness was determined based on surface microgeometry measurements in a two-dimensional (2D) system. The roughness parameters were determined after the measured profiles were leveled. Symmetrical surface profiles were analyzed. An exemplary 2D roughness profile for the CaP coating deposited on the CpTi G4 substrate from the acetate bath at −3 V for 30 min at 20 and 70 °C is presented in Figure 8. The basic parameters of the surface texture were determined based on ISO 4287 [45]. Table 2 shows the determined values of parameters, such as *Pa*—arithmetic mean deviation of the primary profile, *Pq*—root mean square deviation of the primary profile, *Pp*—maximum peak height of the primary profile, *Pv*—maximum valley depth of the primary profile, *Pt*—total height of the primary profile, *Psk*—skewness of the primary profile, *Pku*—kurtosis of the primary profile, *Ra*—arithmetic mean deviation of the roughness profile, *Rz*—maximum height of the roughness profile, and *Rp*—maximum peak height of the roughness profile [45].

The *Psk* parameter values indicate the symmetrical profile of each of the tested surfaces (Table 2). The arithmetic mean of the roughness profile for CpTi G4 after sandblasting is 1.65(7) μm [18], while, for the sandblasted CpTi G4 with the CaP coating deposited at 20 °C, it is 2.39(8) μm, which indicates an increase in surface roughness. With the increase in the deposition temperature of the CaP coatings, *Ra* increases up to 60 °C, for which the *Ra* value is 3.95(12) μm. In the case of the CaP coating deposited at 70 °C, a small decrease in the *Ra* parameter value is visible, which can be caused by a slight leveling of the surface due to the formation of CaP agglomerates (Table 2). Among the determined basic surface texture parameters, the *Ra* parameter best reflects the roughness size on larger surfaces because it eliminates the effect of single, irregular hills or cavities. To local field assessment, *Rz* seems to be the most appropriate parameter. The *Rz* parameter for the sandblasted CpTi G4 takes a value of 11.20(83) μm [18], while the CaP coatings have higher values from 15.10(71) to 23.75(7) μm in the temperature range from 20 to 60 °C, respectively (Table 2). *Rz* determined for the CaP coating deposited at 70 °C slightly decreases. The obtained profilometric results are in agreement with the microscopic observations (Figure 8). Considering that the surface roughness of dental implants with *Ra* between 1 and 3 is required [18], the optimal surface properties are shown by the CaP coating deposited from an acetate bath at −3 V for 30 min at 20 °C.

### 3.4. Effect of Deposition Temperature of CaP Coatings on In Vitro Bioactivity

**Figure 9:**
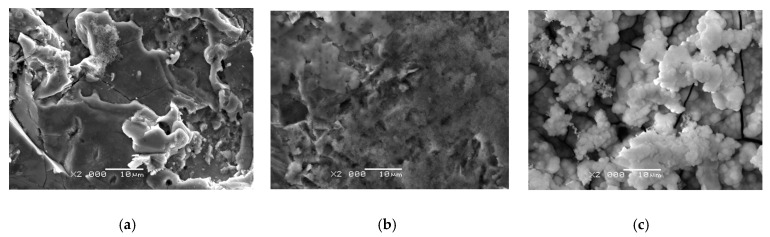
SEM image of the surface of CpTi G4 substrate mechanically polished, sandblasted and sterilized after bioactivity test for (**a**) 1, (**b**) 3, and (**c**) 7 days of immersion in SBF at 36.6 °C (×2000 magn.).

**Figure 10:**
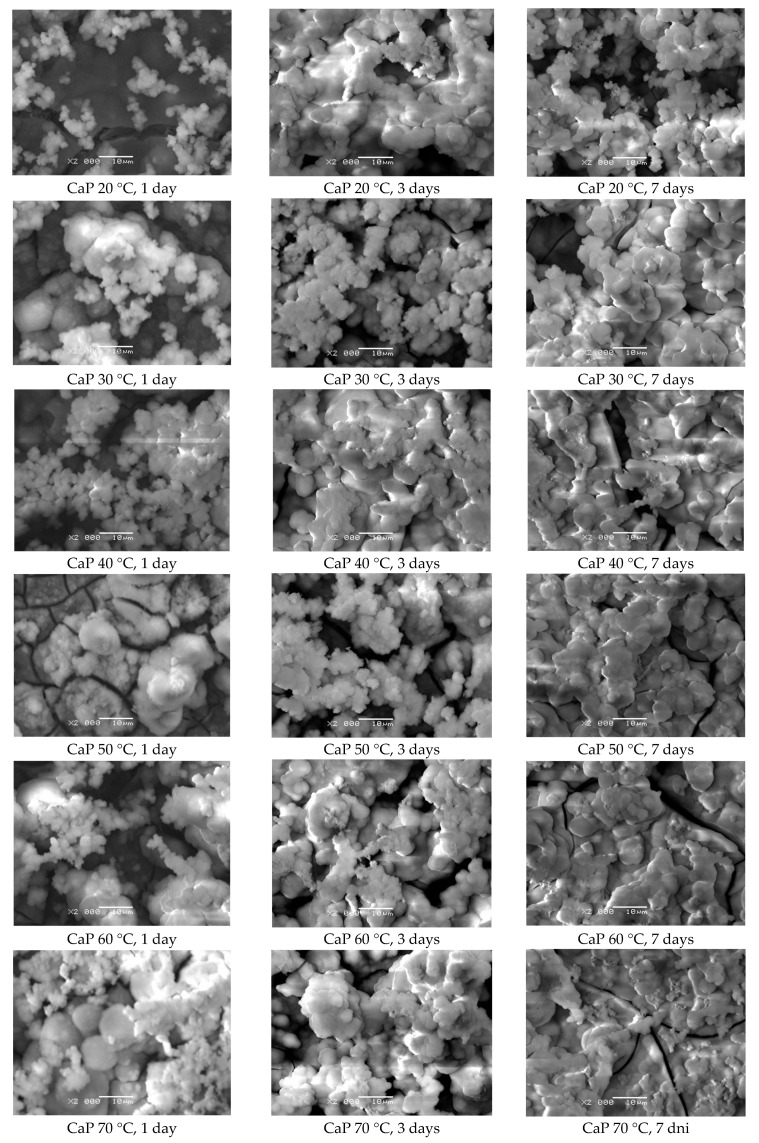
SEM image of the CpTi G4 substrate with CaP coating deposited at −3 V for 30 min, at 20 to 70 °C, after bioactivity test for 1, 3, and 7 days of immersion in SBF at 36.6 °C (×2000 magn.).

The bioactivity of materials is the biological reaction of the tissue to the material. The SBF immersion test method allows us to predict in vivo the ability of a material to bind to bone tissue. Bioactivity tests for 1, 3, and 7 days carried out for the CpTi G4 substrate were successively subjected to mechanical polishing, sandblasting, and sterilization (Figure 9), and with a CaP coating obtained at the potential of −3 V for 30 min in the temperature range from 20 to 70 °C (Figure 10).

All tested materials formed Ap on their surface, but to a different extent (Figure 9 and Figure 10). In the case of the CpTi G4 substrate subjected to mechanical polishing, sandblasting, and sterilization, a very low Ap formation ability was found both after 1 and 3 days of the bioactivity test (Figure 9a,b). Only trace amounts of Ap were observed on the surface of the CpTi G4 substrate. The number and size of the formed Ap crystallites increased after 7 days of immersion in SBF (Figure 9c).

CaP coatings deposited on the CpTi G4 substrate, after 1 day of immersion in SBF, formed significant amounts of Ap on more developed parts of the surface (Figure 10). Numerous spherical agglomerating particles were observed that grew as the test time increased to form a continuous layer of Ap after 7 days of SBF immersion. The ability of bioactive CaP coatings to form Ap increased with the increase in the deposition temperature, which was related to the greater development of the surface of the tested materials. Apatite was fairly evenly distributed over the surface of the CaP coatings, which acted as a precursor in the process of Ap formation in SBF. Increasing the immersion time caused a thicker layer of Ap to build up, which flakes off during the test. Too much Ap formed on thicker CaP coatings may disrupt the induction of natural bone tissue for growth and thus the osseointegration process. The bone defect that occurs during the preparation of the bone bed during the implantation procedure is regenerated while maintaining the original morphology.

The precipitation of carbonated Ap (dahlite) in SBF can be described by the chemical reaction (9) [53]:

(9) $$10{\mathrm{Ca}}^{2+}+6{\mathrm{HPO}}_{4}^{2-}+2H_{2}O\to {\mathrm{Ca}}_{10}{\left({\mathrm{PO}}_{4}\right)}_{6}{\left(\mathrm{OH}\right)}_{2}+8H^{+}.$$

The mechanism of a dahlite layer formation is based on a reduction in the local concentration of calcium and phosphate ions. In contact with osteoinductive biomaterials, calcium and phosphate concentrations decrease in cell culture media due to Ap precipitation [54,55]. Habibovic and co-workers [56] proposed the concept of biological Ap formation based on dissolution and calcium release prior to Ap precipitation, which is excluded in our considerations. It should be noticed that the formation of a dahlite layer on the biomaterial surface is a pre-requisite, but not a determinant for intrinsic osteoinduction [23]. However, it has been reported that an increase in bioactivity in vitro leads to an increase in intrinsic osteoinduction [57,58]. ACP is the natural Ap precursor phase that can be allocated and deposited by intracellular and mineral-containing vesicles at the gaps in the collagen matrix. ACP acts as a transition phase that is readily carried as a precursor to Ap growth [23].

**Figure 11:**
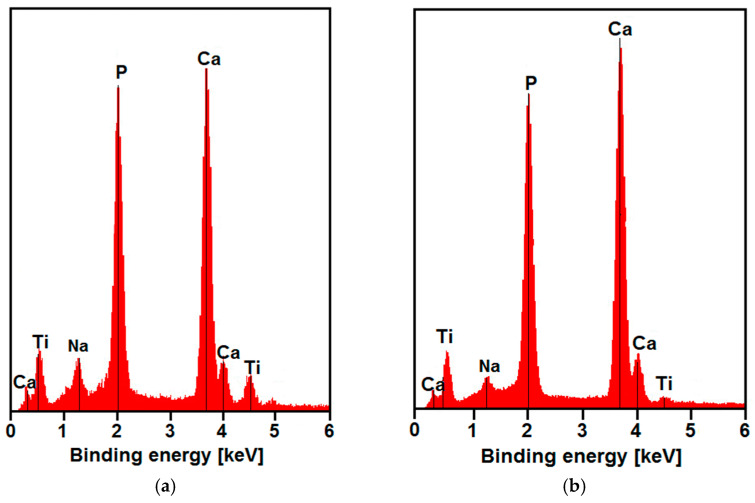
EDS spectrum after 7 days of immersion in SBF at 36.6 °C: (**a**) CpTi G4 substrate; (**b**) CaP coating deposited at −3 V for 30 min at 20 °C.

The analysis of the local chemical composition from the CpTi G4 substrate surface (Figure 11a) and CaP coating deposited at −3 V for 30 min at 20 °C (Figure 11b) after the bioactivity test showed the presence of titanium peaks in the exemplary EDS spectra in both cases. Additionally, the Na-originated peaks whose ions were included in SBF were identified (Table 1). The Ca- and P-derived peaks showed different intensity, which was directly proportional to the amount of Ap formed on the surface of the samples. The element concentration in at.% determined from the EDS spectrum in Figure 11a was 55.52(97) for Ca, 34.26(62) for P, 6.06(32) for Na, and 4.16(42) for Ti. The analysis of EDS spectrum in Figure 11b revealed the following element concentration in at.%: 58.88(99) for Ca, 36.57(63) for P, 1.07(18) for Na, and 3.48(19) for Ti. The EDS results confirmed nucleation and the first Ap formation after 1 day of immersion in SBF. Based on EDS analysis after 7 days of the bioactivity test, the Ca:P ratio was found to be 1.6 for all samples, which means the formation of HA with hexagonal structure [59]. Thus, it can be concluded that the application of ACP coatings on the CpTi G4 surface may have a positive effect on the induction of natural Ap in the form of HA. The presence of such a crystallographic form of calcium phosphate after the bioactivity test indicates that ACP coatings are an excellent material for the production of new bone tissue.

**Figure 12:**
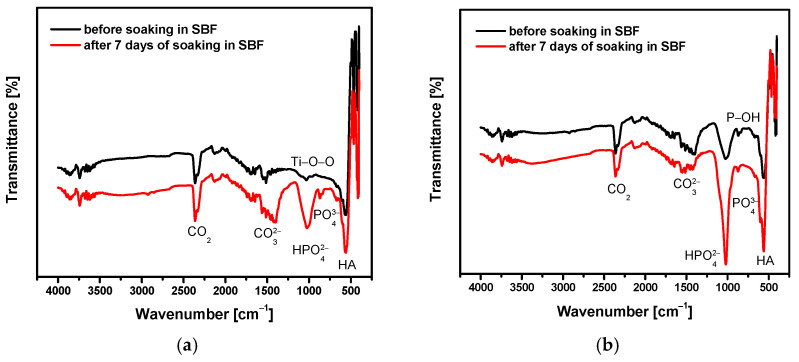
ATR-FTIR spectrum collected after 7 days of immersion in SBF at 36.6 °C for: (**a**) CpTi G4 substrate; (**b**) CaP coating deposited at −3 V for 30 min at 20 °C.

The EDS results were verified by FTIR measurements. The use of FTIR as vibrational spectroscopy allowed for the identification of phases in the studied Ca-PO_4_-H_2_O system, in which the phosphate and hydroxide groups cause very specific vibrations, which are difficult to identify with other methods. An example of ATR-FTIR absorption spectra collected for the CpTi G4 substrate and the CaP coating deposited at −3 V for 30 min at 20 °C before and after 7 days of immersion in SBF at 36.6 °C is shown in Figure 12.

Analysis of the ATR-FTIR spectrum obtained for the CpTi G4 substrate before the bioactivity test showed the presence of a TiO_2_-derived peak at a wavenumber of 571 cm^−^^1^, which is the result of vibrations of the Ti-O-O bond (Figure 12a). The peak at 1646 cm^−^^1^ corresponds to absorbed H_2_ and the peak at 2361 cm^−^^1^ is attributed to the vibrations of CO_2_ absorbed from the air [60]. After 7 days of soaking in SBF, HA can be clearly identified in Figure 12a. All the characteristic absorption bands of HA are present at 1021, 600, and 561 cm^−^^1^, which correspond to the ${\mathrm{PO}}_{4}^{3-}$ and ${\mathrm{HPO}}_{4}^{2-}$ group [61]. A band ranging from 1596 to 1401 cm^−^^1^ corresponds to the ${\mathrm{CO}}_{3}^{2-}$ group [61]. The peak at 3742 cm^−^^1^ corresponds to the O–H bonds, while the peak at 2361 cm^−^^1^ is related to the vibrations of CO_2_ absorbed from the air. Based on the analysis carried out on the basis of the results presented in Figure 12a and SEM/EDS (Figure 9 and Figure 11a), it can be concluded that CpTi G4 subjected to mechanical polishing, sandblasting, and sterilization shows low bioactivity in vitro. The applied surface treatment inhibits the development of natural Ap on the titanium surface.

Figure 12b shows the ATR-FTIR spectrum collected for the CaP coating before and after the bioactivity test for 7 days. Analysis of the FTIR results for the CaP coating before soaking in SBF showed a band at 1024 cm^−1^ (stretching band), and a band at 559 and 465 cm^−1^ (bending vibrations) due to the presence of phosphate groups. A visible peak at 869 cm^−1^ (stretching vibrations) results from the presence of ${\mathrm{PO}}_{4}^{3-}$ ions which are detected for apatites that are ${\mathrm{HPO}}_{4}^{2-}$ carriers due to stretching of the P–OH bond. The obtained ATR-FTIR spectrum is typical for amorphous calcium phosphates [62,63]. This is evidenced by the band in the 600 to 400 cm^−1^ wavelength range which, in the case of ACP, is blurred compared to the classical HA and forms one peak. The ATR-FTIR spectrum obtained for the ACP coating after the 7-day SBF immersion test shows the enhancement of the peaks at 1024, 559 and 465 cm^−1^ corresponding to the group ${\mathrm{PO}}_{4}^{3-}$ and ${\mathrm{HPO}}_{4}^{2-}$ [25]. This proves the formation of more calcium phosphate on the surface of the coating and, therefore, its high bioactivity. The “apatite” nature of the tested ACP coating was confirmed. The obtained results in Figure 12b and SEM/EDS (Figure 10 and Figure 11b) confirm the high in vitro bioactivity of the obtained CaP coatings.

## 4. Conclusions

CaP coatings were successfully deposited on the CpTi G4 substrate subjected to mechanical polishing, sandblasting, and autoclaving using the ECAD method from the acetate bath at the deposition potential −1.5 to −10 V relative to the open circuit potential for 10 to 60 min using the bath temperature from 20 to 70 °C.

Chronoamperometric characteristics of the ECAD of the CaP coatings showed that rising deposition potential and bath temperature increased both the current density modulus and the amount of the deposited CaP due to the increase in the deposition rate. The surface morphology of the CaP coatings was significantly changed with deposition potential, time, and temperature. EDS and FTIR analysis confirmed that ACP coatings were deposited on the titanium surface. The thickness and surface roughness of the ACP coatings were increasing functions of the deposition potential, time, and bath temperature.

The ECAD mechanism of ACP coatings on the CpTi G4 substrate in the acetate bath was based on three stages. The first stage was assigned to the reduction in oxygen, the second stage corresponded to the reduction in phosphates, and the third stage was associated with the reduction in water. A local increase in pH at the cathode surface was due to cathodic polarization, leading to OH^−^ ions formation. The Ca^2+^ ions migrated from the bath to the negatively charged cathode surface and reacted with the ${\mathrm{PO}}_{4}^{3\;-}$ ions, resulting in ACP synthesis on the cathode surface.

The ACP coatings obtained under the proposed conditions showed increased bioactivity in SBF under in vitro conditions compared to the CpTi Grade 4 substrate, which increased with coating deposition temperature.

The optimal ECAD-derived coating for dentistry was deposited from the acetate bath at −3 V for 30 min at 20 °C. This ACP coating was characterized by the thickness of 11.3(7) μm, *Ra* of 2.39(8) μm, and showed the ability to form the bone-like Ap in SBF.

## Figures and Tables

**Table 1:** Composition of the SBF solution used for the test of the bioactivity of the tested materials.

Ion Type	Concentration [mM]
Na^+^	142.0
K^+^	5.0
Mg^2+^	1.5
Ca^2+^	2.5
Cl^−^	147.8
${\mathrm{HCO}}_{3}^{-}$	4.2
${\mathrm{HPO}}_{4}^{2-}$	1.0
${\mathrm{SO}}_{4}^{2-}$	0.5

**Table 2:** Basic surface texture parameters with standard deviations (*SD*) for the CaP coatings deposited on the CpTi G4 substrate from the acetic bath at −3 V for 30 min at the bath temperature of 20–70 °C, according to ISO 4287 [45].

Parameter	20 °C		30 °C		40 °C		50 °C		60 °C		70 °C	
	Value	*SD*	Value	*SD*	Value	*SD*	Value	*SD*	Value	*SD*	Value	*SD*
*Pa* [µm^2^ m^−^^1^]	2.98	0.15	3.83	0.07	4.41	0.03	4.37	0.20	4.65	0.31	3.94	0.01
*Pq* [µm]	3.69	0.22	4.78	0.04	5.53	0.05	5.41	0.26	5.87	0.35	4.89	0.01
*Pp* [µm]	9.62	0.69	13.85	4.03	23.30	3.68	16.90	1.13	16.35	0.21	16.25	0.49
*Pv* [µm]	11.05	1.06	15.35	0.92	15.00	0.71	16.05	1.48	18.20	0.42	12.80	0.28
*Pt* [µm]	20.65	0.35	29.20	3.11	38.20	4.38	32.95	0.35	34.55	0.21	29.05	0.21
*Psk*	−0.10	0.30	−0.10	0.17	0.15	0.01	0.07	0.09	−0.01	0.04	0.22	0.07
*Pku*	2.70	0.04	2.95	0.28	3.33	0.33	2.70	0.01	2.89	0.17	2.82	0.01
*Ra* [µm]	2.39	0.08	3.46	0.02	3.67	0.16	3.74	0.16	3.95	0.12	3.48	0.08
*Rz* [µm]	15.10	0.71	20.95	0.78	22.30	0.28	23.25	1.06	23.75	0.07	20.50	1.70
*Rp* [µm]	7.21	0.33	9.94	0.80	11.55	0.49	11.85	0.21	11.60	0.42	10.57	0.90

## Data Availability

Not applicable.
